# Critical Reflections on the COVID-19 Pandemic from the NHS Frontline

**DOI:** 10.1177/13607804231156293

**Published:** 2023-02-22

**Authors:** Anthony Lloyd, Daniel Briggs, Anthony Ellis, Luke Telford

**Affiliations:** Teesside University, UK; Universidad Europea de Madrid, Spain; University of Lincoln, UK; University of York, UK

**Keywords:** COVID-19, lockdown, NHS, social harm, work

## Abstract

The COVID-19 pandemic fundamentally changed the way we live, work, and interact with each other. Nowhere was the pandemic more profoundly experienced than on the frontline of healthcare. From overwhelmed Intensive Care Units to shortages of Personal Protective Equipment (PPE) and clap for carers, the UK’s National Health Service (NHS) became the focal point for the pandemic response. Utilising data from online survey responses (N = 16) complemented by four online interviews and one face-to-face interview (N = 5) with NHS workers primarily during the height of the pandemic, this article offers a preliminary analysis on the challenges the UK’s healthcare workers faced through working in conditions of crisis management. The article particularly addresses NHS workers’ amplification of fear, anxiety, and exhaustion; the absence of widespread solidarity; and implications of the absence of coherent governmental messaging upon the workforce. We situate this discussion within a critical account of neoliberal political economy, the theoretical framework of social harm, and the absence to explicate the harmful conditions of the pandemic’s frontline. While the data are confined to the UK’s NHS workers, its findings are relevant to other countries across the world that enacted similar responses to deal with COVID-19.

## Introduction

The COVID-19 pandemic interrupted the normal rhythms of social life around the world (Briggs et al., 2021). Following the evident pressure on the Italian healthcare systems in February 2020, governments across the globe enacted a range of non-pharmaceutical interventions (NPIs). Designed to curb the spread of the virus and protect healthcare systems from being overwhelmed with demand, these public health measures included social distancing, mask wearing, contact tracing, and lockdowns (Ferguson et al., 2020; Miles et al., 2021). This reshaped living and working conditions, limited social interaction, and restricted the citizenry’s movement (Briggs et al., 2021a). In the UK, the government initiated a first lockdown on 23 March 2020, which was initially announced as a 3-week measure to ‘protect the NHS’, though it lasted until June 2020. Various restrictive measures were implemented at varying grades of intensity for around 2 years, with huge implications for the citizenry, particularly for those working within the NHS.

The NHS was at the centre of the medical and governmental response to the COVID-19 pandemic and the political and economic fallout (Logan, 2021). For 10 weeks during the first UK lockdown, the nation ‘clapped for carers’ every Thursday night in recognition of NHS employees (Wood and Skeggs, 2020). While in early 2021 the government’s proposed 1% pay increase for NHS staff was met with anger and hostility given their efforts during the pandemic (BBC, 2021), a revised summer offer of 3% was also met with criticism (Campbell and Allegretti, 2021). As Newman et al. (2022: 778) indicated, the pandemic was a profoundly difficult time for NHS staff who felt ‘distress and uncertainty’ ‘as they felt enormous burden to adequately complete their professional, personal and civic responsibility to keep everyone safe’.

Drawing upon data collected via online surveys, four online semi-structured interviews, and one face-to-face interview with NHS workers across the UK primarily throughout the pandemic, this article offers unique insights into the challenges they faced. It is organised as follows. After discussing the NHS before the pandemic, the article presents the methodology and theoretical framework. Next, the findings are structured into four sections including (a) emergency response: implications for care, (b) cumulative weight of working through the pandemic, (c) absent solidarity, and (d) absence of coherent government responses. Therefore, the article offers a preliminary analysis on the difficulties NHS workers endured during this unique historical period, including the intensification of exhaustion and the absence of stability and protection, laying the groundwork for further sociological research.

The article ties the micro-level experience of nurses, doctors, pharmacists, and other NHS personnel with the macro-level of political economy. The focus on certain staff groups reflects our convenience sample and while we do not mention social care, we have specifically addressed the experience of adult social care workers elsewhere (Briggs et al., 2021c). Theoretically, we utilise elements of a zemiological/social harm framework (Hall and Winlow, 2015; Lloyd, 2018; Raymen, 2022; Telford and Lloyd, 2020), which we unpack in more detail in the ‘Methodology’ section. This allows us to both analyse the social harms of working on the pandemic’s frontline and consider the probabilistic harmful outcomes of absence. Issues concerning social relations and cohesion have been evident throughout the pandemic and the experience of NHS staff offers an insight into solidarity, social antagonism, and harm. While this is a preliminary analysis since the findings are drawn from a relatively small sample size, they are relevant to countries around the world which possess similar healthcare systems that have witnessed organisational restructuring under neoliberalism and endured COVID-19 restrictions, particularly lockdowns (Becque et al., 2022; Briggs et al., 2021; Newman et al., 2022). Before we address the experiences of NHS staff, we start with a brief contextual discussion of the NHS to understand the working conditions leading in to the pandemic.

## The NHS before the pandemic

The NHS was created in 1948 with universality at its centre; regardless of their ability to pay, each UK citizen was entitled by right to free healthcare (Pollock, 2004; Webster, 2002). The NHS was centrally funded via taxation but operated at the regional and district levels, with responsibility for local healthcare needs (Pollock, 2004). This represented the political economic philosophy of social democracy during capitalism’s post-war period (Harvey, 2005), whereby sustained government intervention ensured a balance between state and market (Hunter, 2008). The shift towards a neoliberal free-market political economy in the late 1970s represented a significant change in the organisational philosophy (Hunter, 2008). The growth of ‘New Public Management’ under Conservative governments (1979–1997) and ideological continuation under the ‘Modernisation’ project of New Labour (1997–2010) injected business principles into the NHS, while opening the health sector to market forces through outsourcing and competition (Davis et al., 2015; Leys and Player, 2011; Pollock, 2004). While from 2010 onwards austerity was ostensibly a belt-tightening exercise to reduce public spending, it arguably represented the prioritisation of markets over democracy (Streeck, 2016). In a climate of cost-cutting and efficiency savings (Carter, 2016), the NHS’s ability to maintain appropriate standards of care for patients was tested (Campbell, 2016) long before the emergence of the COVID-19 pandemic.

As the largest employer in England with an estimated 1.3 million employees (NHS England, 2021), the political economy plays a significant role in shaping working conditions. Two-thirds of the expenditure from constrained budgets goes on workforce (NHS Employers, 2017a), with around 70% of recurring costs relating to staffing (Imison and Bohmer, 2015). Therefore, issues around staff well-being and workforce composition have direct consequences on patient care and outcomes. Recent research identified problems related to staff recruitment, well-being, and retention (Crawford et al., 2015; Imison, 2016; NHS Employers, 2017a; The Smith Institute, 2015). As of June 2020, the NHS had over 83,000 FTE vacancies across all staff groups (Buchan et al., 2020), while 89% of NHS Trusts reported in 2015 that they use agency and temporary staff to meet staff shortages (The Smith Institute, 2015). The consequences of over-reliance on temporary staffing include higher wage bills, poor continuity of care, and lower staff morale (Imison and Bohmer, 2015; Kirkpatrick and Hoque, 2006).

Evidently, recruitment, well-being, and retention are vital components in the NHS’s ability to work within constrained budgets and maintain effective standards of patient care. Low morale affects recruitment, staff well-being at work, and the capacity to retain employees. Low pay often compels staff to seek alternative opportunities – for instance, one study found around 8% of nurses who left an NHS Trust did not transfer to another NHS organisation (Imison and Bohmer, 2015), while a different Trust reported 40% of staff turnover came from employees who leave in the first year of their employment (NHS Employers, 2017a). According to the 2020 NHS Staff Survey, more than one in four employees admitted they often think about leaving the organisation (NHS England, 2021). Prior to COVID-19, nurses who were leaving the register were reporting that workload, job stress, and mental ill health were the main reasons for leaving (Buchan et al., 2020). Reliance on bank and agency staff increases the fiscal cost to an organisation, while overseas recruitment of qualified and experienced nurses and clinicians ease shortages (Tailby, 2005; The Smith Institute, 2015). Within this context, staff sickness rates reached their highest level in a decade in July 2019 as more than 1.5 million full-time equivalent working days were lost to sickness absence (NHS Digital, 2019).

This brief contextual discussion highlights the main issues within the UK’s NHS before the COVID-19 pandemic. The impact of the pandemic on NHS staff must be considered in this context as the virus did not arrive into a vacuum. Before we consider the intensified challenges NHS workers faced during the pandemic, the following section outlines the methodology behind the larger global study that this article’s data are deployed from.

## Methodology

Data in this article are derived from a multi-stage, global, mixed-methods project (see Briggs et al., 2021a for a full account). The study’s aim was to capture people’s experiences of and feelings about the pandemic as it unfolded. In line with Rhodes and Lancaster’s (2020) suggestion that public health emergencies should be studied as they unfold, we set out in March 2020 to capture this unique and epochal period as it progressed. It involved five phases:

- Phase 1 – Lockdown; approximately March–May 2020
- Phase 2 – ‘New normal’; approximately June–September 2020
- Phase 3 – ‘Viral hiatus’; approximately October 2020–March 2021
- Phase 4 – ‘Hindsight’; approximately April 2021–October 2021
- Phase 5 – ‘Vaccine endgames’; approximately November 2021 (ongoing).

These topics were shaped by the data that we collected, empirically illuminating the pandemic’s key stages. Utilising quantitative and (distanced) qualitative methods, so far, we have gathered 2923 survey responses, 120 hours of digital ethnography, 57 online interviews, and engaged in traditional ethnography when restrictions permitted. The online survey contained open and closed questions about participants’ experiences of the pandemic. It was advertised on social media, such as LinkedIn and COVID-19 Facebook forums, and generally took around 15 minutes to complete. While the semi-structured survey did not ask specifically about healthcare staff, we received 16 in-depth responses from NHS employees. By asking at the end of the survey if anybody would be interested in being interviewed, we managed to undertake four online, face-to-face interviews with NHS staff on Zoom, and obtained nuanced opinions on working in the NHS in real-time during the pandemic. Interview questions revolved around participants’ life before and during the COVID-19 pandemic and how it had impacted upon them. We also include one face-to-face interview with another NHS worker in this article’s methodology from a different sociological study that one of the co-authors recently worked on, enhancing the article’s robustness. This project explored the social problems in the UK’s ‘left behind’ places, with employment conditions being a key focus (see Telford and Wistow, 2022). Indeed, thematic analysis of the above data sources revealed codes and themes pertinent to UK healthcare workers.

While there is no consensus on what constitutes an adequate qualitative sample, Baker and Edwards (2012: 8) suggest ‘a small number of cases, or subjects, may be extremely valuable and represent adequate numbers for a research project’. At the time that most of the data were collected, there was a lot of caution among NHS workers about speaking to anyone particularly out of fear of losing their jobs. Given the COVID-19 restrictions and fear among workers, we felt this was the most sensitive and open way to approach our study. These NHS respondents’ occupations include nurses, doctors, a psychologist, HR officers, a healthcare assistant, and hospital pharmacy employees. They encompass both males and females and they range in age from the twenties to sixties.

As this sample was primarily drawn from the COVID-19 project, a convenience sample was utilised. This article’s methodology contains a relatively small sample size, meaning the article’s findings are not universally replicable. However, it can offer analytical generalisability (Telford and Lloyd, 2020). This is where the findings are corroborated or problematised through further research. As mentioned, scholarship on the NHS pre- and post-pandemic also highlighted workplace stress, burnout, staff absences, fear, and a shortage of PPE (Becque et al., 2022; Crawford et al., 2015; Johnson et al., 2003; Newman et al., 2022). Moreover, the multi-method approach during the pandemic offers unique, real-time insights into healthcare workers labouring on the pandemic’s frontline, which few sociological studies can offer. Indeed, the research aims are modest – to offer a preliminary analysis and further evidence on the harms of working in the NHS during the pandemic – encouraging further sociological research.

Data for the global COVID-19 study were collected by the lead researcher who works in Spain, where formal ethics procedures are not mandated. Rather, there is a scholarly obligation to embed general ethical principles in research (see European Commission, 2018). These generally mirror the ethical guidelines stipulated by the British Sociological Association (BSA), meaning the research was conducted in accordance with the BSA’s (2017) Statement of Ethical Practice. Therefore, all places are anonymised and respondents pseudonymised. A participant information sheet was administered for both the online survey and interview participants, describing the study, its aims, intended data usage, confidentiality, and their rights as respondents. Participants were given the right to not answer certain questions and to withdraw at any time from the research. At the start of the online survey respondents were given a box to tick, which indicated their informed consent to participate. Interviewees gave recorded verbal consent after reading the information sheet, with the interviews generally lasting between 60 and 90 minutes. Throughout data collection, participants were interviewed sensitively and empathetically, drawing on the research team’s sociological research experience. Participants were eager to tell their stories to someone, with some stating that it felt therapeutic to share their experiences during a turbulent and unprecedented time. Regarding the interview from the ‘left behind’ places study, it was conducted in August 2022; it lasted 35 minutes and was audio-recorded; a participant information sheet and consent form were administered, and the work received institutional ethical clearance.

The four themes presented shortly speak of the experience of NHS workers since the onset of the pandemic. Among other issues, they expose the intensification of fear, stress, and the absence of stability and emotional well-being. Collectively, these themes raise important questions about workplace harm, social cohesion, and the relationship between macro structures and micro experience. The article now briefly presents the theoretical framework before explicating the empirical findings.

## Zemiology/social harm

Emerging in the 1990s, Zemiology is concerned with studying social harms, especially the negative consequences of legal decisions and behaviours of governments (Boukli and Kotzé, 2018; Canning and Tombs, 2021; Hillyard and Tombs, 2004). Based upon the ancient Greek word *Zemia* which means to injure, harm, or cause damage, Zemiological scholars have explored the normalised and embedded harms of neoliberal political economy and cultures, workplaces, global corporations, and states (Boukli and Kotzé, 2018; Canning and Tombs, 2021; Lloyd, 2018; Pemberton, 2016; Raymen, 2022; Yar, 2012). While we acknowledge debates within the social sciences over identifying precisely what social harm means (Canning and Tombs, 2021; Pemberton, 2016; Raymen, 2022) – in part to avoid the concept becoming a catch all phraseology that loses its conceptual utility – this article utilises Hall and Winlow’s (2015) interpretation of social harm.

This conceptualisation uses the negative motivation to harm, that is, the unintended consequences of the normal functioning of our political economic system, as well as the positive motivation to harm which embodies the subjective willingness to inflict harm upon each other (Hall and Winlow, 2015). While the presence of phenomena possesses probabilistic causal tendencies, for Hall and Winlow (2015) and Lloyd (2018) absence also possesses probable causal tendencies. Other scholarly work, for instance, attested that the absence of economic security, mental well-being, and a sense of dignity from employment can have harmful impacts upon one’s life (Lloyd, 2018; Telford and Lloyd, 2020). As we will now see, for NHS workers this includes an absence of protection, security, and stability which may correspond to harmful outcomes including mental distress. In contemplating the probabilistic causal tendencies of absence, this framework allows us to situate the experiences of NHS workers throughout the pandemic in the context of an absence of protection and stability (Lloyd, 2018).

## Findings

### Emergency response: implications for care

As the first lockdown was implemented on 23 March 2020, working conditions within the NHS changed dramatically. While the lockdown was announced as a three-week measure to protect the NHS, it lasted until 1 June 2020. This was accompanied by a 10-week clap for carers campaign every Thursday night in recognition of the work of the NHS and other key workers. In our Phase 1 survey, an HR officer within an NHS trust felt: ‘the NHS has galvanised quickly to put in place emergency measures’. Crisis management practices were implemented, and organisational change occurred to manage the first wave. Some respondents noted an energised positivity as an ‘all-hands-on deck’ siege mentality emerged. This required the redeployment of staff to different roles, additional duties, and the use of overtime. Rachel, female, 38, NHS doctor, indicated how the ‘hugely challenging’ crisis management response meant everyone pulled together to reconfigure operations and manage the crisis. However, the interview from the ‘left behind’ places study with Danielle, 28, a healthcare assistant on an acute medical ward, described the impact of the first wave in terms of reconfigured working practices. This served to heighten tension and fear in an already stressful working environment:

We had people on end-of-life care, literally their last few days, and we had to tell their families they weren’t allowed in. During the pandemic, we had to forget our training and before we did anything we had to put PPE on. Even when somebody was having a cardiac arrest; you couldn’t go to that patient until you had PPE on.

The human suffering of not allowing end-of-life patients to see loved ones before they died during the pandemic amounted to what Becque et al. (2022: 775) cast as ‘restricted farewells’, denying patients ‘meaningful-end-of-life moments’. This was difficult for NHS workers like Danielle, claiming it ‘was hard sticking by your argument even when I didn’t believe in it’. Relatedly, Katie, female, 43, a hospital pharmacy worker, spoke about the detrimental impact of disruptions to patients picking up prescriptions from the hospital pharmacy, though she acknowledged challenges with this service prior to COVID-19 which she attributed to austerity. When measures were introduced to mitigate the spread of COVID-19, the pharmacy had no courier system in place to deliver medication to patients off-site. While volunteers helped, pharmacy staff would finish long shifts in the hospital and then deliver medications to patients at home. The impacts on the workforce, Katie explained, were huge: ‘I’d say 25% of our workforce are not working and they were already under pressure, so Covid has made it worse’. The negative motivation to harm reflects the unintended consequences of neoliberalism’s normal functioning (Hall and Winlow, 2015). Neoliberalism has been described as the most harmful form of political economy (Pemberton, 2016) and the decade of austerity imposed upon the NHS stripped back capacity and placed additional pressure on a reduced workforce. This meant staff routinely worked overtime during the pandemic to ensure work was completed, stress and tension increased significantly, and sickness absence rose, embodying an absence of stability from the workplace.

The impact of the emergency response also meant public hesitancy to seek medical intervention for non-COVID-19 related conditions that often required urgent care. In the UK, non-essential and routine services were cancelled or postponed in a strategy that could accurately be described as ‘Covid-19 above all else’. Staff were not prepared for the impact:

While we did our preparation, we thought people would still come into the hospital with the normal stuff but actually because the message had been “stay at home, protect the NHS” I think in hindsight this was aimed at enforcing the lockdown rules on people. But, we didn’t see all the normal stuff coming in. This became worrying. The messaging was so strong. It became so true for people like if you died from Covid, society had let you down. People haven’t been following the rules. (Rachel, NHS doctor)

Rachel argues that heart attacks, strokes, delayed cancer diagnoses, as well as an increase in alcohol-related problems, while perhaps not preventable, were treatable. However, in her experience, people stayed away due to government messaging and fear of the virus. Interviewed during the UK’s second wave (approximately September 2020–March 2021), she indicated that new COVID-19 cases were piling on top of the ‘stuff which we didn’t deal with in the first lockdown’. With hindsight, she believed it was dangerous to not keep other services going and that some determinations as ‘non-essential’ may have proved to be wrong (see Hamilton, 2020). This then created a vicious feedback loop that will continue to place pressure upon the under resourced health service in the future. A significant backlog of non-essential work combines with higher levels of interventions and treatments needed for medical issues that would have been dealt with at an earlier stage before the pandemic. This amplifies workplace pressure and workloads and thus the absence of stable and secure working conditions. As one NHS consultant wrote in March 2021, while ICU beds allocated to COVID-19 patients were falling, those beds were being quickly filled by a range of other patients, including some who would not have required intensive care had they sought medical assistance sooner (Corrigan, 2021).

By summer 2021 an estimated 5,000,000 people were on NHS waiting lists, the highest number on record (Campbell and Duncan, 2021). The backlog and pressure from *all other health conditions* became part of the political discussion about the UK’s restrictions. However, many respondents highlighted how pressure on the NHS came not from high numbers of COVID-19 patients but significant staff absences through self-isolation, sickness, and practical issues. This was elucidated by Danielle:

There was a lot of staff off who had Covid, and then you had other factors like nurseries and stuff shutting so people struggled for childcare. If people had other conditions like asthma, then they were often off, so there was a lot of overtime and short shifts. There was a lot of staff absences. . .When staffing levels rose, we could give them [patients] proper care.

Sickness absence through adverse physical and mental well-being, employee withdrawal, and heightened vulnerability of harm to COVID-19 represent the consequences of ongoing exposure to workplace harm and the absence of stable working conditions. According to an NHS Providers report, 99% of trust managers were ‘extremely or moderately concerned about the current level of burnout across the workforce’ due to the impact of the pandemic (Buchan et al., 2020: 10). Data presented above appear to corroborate these fears. From a social harm perspective, there is an absence of protection and stability for NHS workers who effectively worked in ‘crisis mode’ for nearly two years, with evident harmful effects. We also see an absence of protection for patients who face long waits and uncertain futures following the decision to suspend all non-essential treatments and surgery as part of the pandemic response. These conditions combined to produce a cumulative weight that was difficult to labour under during the pandemic.

### Cumulative weight of working through the pandemic

When asked about her views on the outbreak of the virus in the context of working in the NHS, Pharmacy Worker, Katie, was to the point: ‘Scary as shit’. That fear encompassed her working life, fears for her children, their futures, and the growing antagonisms that we will report in the following section. Regarding managing work and life throughout a public health crisis, Katie revealed that panic buying, widely reported in UK supermarkets at the start of the pandemic, made shopping difficult after long and exhausting shifts. The absence of face-to-face contact with her patients was also upsetting. Such an absence led Katie to report increased anxiety among pharmacy patients, particularly cancer patients. Another frontline worker, Louise, female, 28, nurse, also felt the start of the pandemic was frightening because ‘it was unknown’. However, Louise argued that she was young, fit, and healthy, meaning that once more was known about the virus, going to work became less problematic and she just got on with it.

The siege mentality noted previously at the outset of the pandemic had seemingly evaporated by the second wave in Autumn 2020. Disappointment at the need for further lockdown measures heightened anxiety and thus the absence of mental and emotional well-being among already burned-out staff. Rachel indicated that staff had not been able to take holidays or recover from the first wave before the second wave hit. Morale was low and fatigue was increasing. Crucially, she argued that the second wave was compounded by the inadvertent consequences of lockdown. This included staff self-isolating after positive COVID-19 tests, staff sickness through mental ill health, and operational problems such as cancelled training during the first wave that impacted on rotas during the second wave. Our data indicated that the impact of working in hospitals through the successive COVID-19 waves within a neoliberalised climate of diminished resources, understaffing, and low morale (Carter, 2016; Newman et al., 2022) produced harmful outcomes that could be defined as a negative motivation to harm:

I felt isolated, alone and increasingly anxious at work’. (NHS nurse, female, 40–49, Phase 1)

Going out a little, back to work as a nurse in community, going into houses, but very scared I’ll bring it home to my family. (NHS Community nurse, female, 60–69, Phase 2)

These responses are indicative of the stress caused by both the virus and the *cumulative weight* of working through the pandemic, with the latter embodying a negative motivation to harm with conditions in the NHS unamenable to workforce stability. Anxiety was heightened out of fear of catching COVID-19 through one’s work, alongside the pressure of working in a prolonged state of crisis mode. Such a dynamic was illuminated by Danielle, who when asked about working through the pandemic, said: ‘it was horrific, absolutely awful’. Ultimately, the NHS workers in our sample are people too; they have been on the frontline of the first global pandemic in a century and an unprecedented set of public health restrictions on our lives (Briggs et al., 2021b), generating harmful outcomes like the absence of mental and emotional well-being. Pharmacy worker, Katie, reported a sense of failure among her colleagues when the UK government enacted its second lockdown in November 2021:

Disappointed, sad, cross because we are all having to double down and lockdown again. Last month until now my anxiety is through the roof. The second wave is here. It is putting us all under immense pressure, more people are getting covid, they are ill, people are affected by the lockdown, they are off work for mental health and then you see people not distancing and just doing what they want.

The impact on the NHS was significant and this was a workforce that had been in crisis mode for almost 12 months. It is little surprise that our third survey, launched at the start of this peak, received no responses from NHS workers. If social harms are visible in the absences around us, then the absence of protection, mental and emotional well-being, and a positive workplace environment are palpable. Workplaces under crisis management are designed to manage the kinds of pressures generated by the virus; however, this comes at a considerable cost to staff who are burned-out and anxious as a result. By the nature of certain forms of work, some jobs increase proximity to harm including healthcare workers, but some harms are avoidable and should be mitigated as far as possible. As mentioned, staff shortages were endemic in the NHS pre-COVID-19, and the respondents’ experience of the pandemic had done nothing to alleviate that. In this sense, NHS workers were left unprepared to cope with a pandemic due to the unintended consequences of government action (and inaction), which was heightened by how initial applause for NHS workers quickly gave way to further division.

### Absent solidarity

As mentioned, throughout the pandemic the NHS was at the forefront of public display. The clap for carers in the initial lockdown (Wood and Skeggs, 2020); Captain Tom Moore’s £100 million fundraising; supportive signs and banners in windows and calls for an ‘NHS Recognition Day’ (Wood, 2020) all indicate appreciation for the work undertaken by healthcare workers. However, our data indicated that public sentiments towards NHS employees were more nuanced. Louise’s perspective, for instance, demonstrated how the initial solidarity and support during the first wave quickly became absent:

In the beginning we got a lot of respect from people. Like, people really noticed us and made the effort to let us know how much they appreciated us. Like we got out of the car one time and some people who were walking past and they stopped and started clapping us, people were genuinely really appreciative at times. It was lovely, some people in the community made us visors and masks because they knew we had a shortage. It was really heart-warming to know people cared, we were all really touched by it. But most people forgot about what we were doing very quickly I think. People go back to how they were before don’t they and they just forget. I remember I went to the supermarket and there was quite a big queue to just get inside and they were allowing NHS and other key workers to jump the queue. So, I went to the front of the queue with my NHS staff card and they let me in, but loads of people in the queue started shouting and saying things like ‘why should she get in without queuing just cos she works for the NHS?’.

The language around the NHS as heroes placed its workers at the forefront of displays of gratitude and affection. However, neoliberalism has crystallised individualism and competition at the heart of political economy and culture (Harvey, 2005). Community and social bonds have broken down and atomisation has grown; some argue that we live in a post-social society (Winlow and Hall, 2013). Society has fragmented into myriad identities and interest groups at the expense of a universal (Raymen, 2022), meaning widespread solidarity became absent and new antagonisms emerged around the continuation or abatement of lockdown, vaccines, and face masks. We do not suggest that people suddenly became anti-NHS. Rather, neoliberal political economy’s value system quickly subverted the initial burst of community spirit, embodying a negative motivation to harm. As an NHS doctor’s survey response suggests:

I think we have a chance as a society to take stock and appreciate what is important. However I think once this is over all will be forgotten and our old habits will continue (I include myself in this). (NHS doctor, male, 30–39, Phase 2)

Recognition works both ways: public attitudes towards NHS staff are complemented by NHS staff attitudes towards the public, particularly those who appear to deny the severity of the virus or defy lockdown restrictions. The survey response below further revealed concerns among NHS staff about those who defied lockdown restrictions and social distancing mandates:

I feel they [people] are playing it down which is scary as the rise is here already and people are still dying with the virus, no vaccine as yet, and people have forgotten already how serious this is and go about like it’s nothing but it’s not. (NHS Community nurse, female, 60–69, Phase 2)

Some respondents argued that complacency and disregard for the rules was harmful. NHS staff were, in some respects, sacrificial offerings, along with other key workers, to keep society functioning (Ellis et al., 2021). The stress, burnout, and emotional consequences of working on the pandemic’s frontline had been undertaken by NHS staff who believed their efforts were negated by the selfish actions of those unwilling to follow the rules. Embodying a positive motivation to harm, it is possible to frame this as a perception of what Hall (2012) refers to as special liberty among those willing to break the rules. In effect, they act outside of the law to further their own instrumental or expressive ends regardless of the harmful consequences to others. Such absent solidarity breeds resentment from those who have, by virtue of their employment, sacrificed themselves, faced increased risk of catching the virus, and suffered physical and emotional strain. This points to a further erosion of social bonds and a new antagonism. Those unwilling to follow the lockdown rules – whether that be politicians breaking rules to enjoy illicit romantic encounters or people acting like COVID-19 is ‘nothing’ – perhaps declare themselves above the rules and free to do as they please, regardless of the harms to others. The angry responses of healthcare workers asked to sacrifice for the greater good appears to show a recognition of the special liberty enacted by others and creates further harm in an already fractured neoliberal society. While the findings have so far documented the pandemic responses and their harmful implications for care, cumulative weight of labouring during the pandemic, and the absence of solidarity, the final ‘Findings’ section presents discontent around the absence of coherent governmental messaging.

### Absence of coherent government responses

Some respondents praised the rapid NHS mobilisation in the first wave but were critical of the government. In response to our first survey, an NHS nurse, female, 60–69, Phase 1, criticised the government for being unprepared, reacting too late, and not getting on top of the crisis. An NHS psychologist, male, 30–39, Phase 1, reported that the government’s mistakes extended to the availability of PPE and that they ‘have often lied about its availability’. While Danielle suggested: ‘We were lucky as we had enough PPE, but I know a lot of the private settings struggled to get it’, Louise outlined how:

The PPE was very difficult to access at first, there was literally nothing for us and they (management) were like insisting we wore it but there wasn’t hardly anything, we had to ration it. At the time, the protocol was that we had to re-wear our PPE, rather than dispose of it after seeing patients. But protocol kept changing and we thought we were safe re-using it, because that was the guidance, and then they’d just suddenly tell us that it wasn’t actually safe and protocol would change. Nobody knew what they were doing. Some of my colleagues were very frightened of the virus but also because of the lack of PPE.

Neoliberalism’s negative motivation to harm is evidenced here. Available bed space, intensive care wards, ventilators, PPE, and staff were limited by a neoliberal regulatory regime that relied on offshored just-in-time production and distribution, leaving it unable to respond quickly (Jones and Hameiri, 2022). An HR officer for an NHS trust, female, 40–49, Phase 1, argued that the government could have taken decisions earlier to enact stricter controls. Other criticisms from NHS staff including a doctor, Phase 2, concerned messaging and transparent communication. Seemingly random decisions and a range of mixed messages were felt to have left the public both confused as to what they could or should do, and more willing to disregard what they viewed as the absence of clearly articulated messages. This was particularly true during the summer of 2020, where a localised four-tier system – with one signifying medium alert and four staying at home – was imposed and restrictions were somewhat eased for many people:

We were encouraged to eat out to help out but then the second wave came and people just went back to normal. So people think ‘we locked up and it came back’ then ‘we went back to normal and it came back’. People are getting bored and found ways around it during the first lockdown. There was a guy in the Co-op the other day who got abusive because he was told he could only have 4 toilet rolls. I feel there is a lot of frustration. It was never really enforced properly. I mean playgrounds are supposed to be closed but everything around where I am is heaving with kids. (Katie, Hospital pharmacy worker)

This was also addressed by survey respondents, including one doctor in Phase 2, who felt stricter enforcement of public gatherings and nuisance behaviour was necessary. Meanwhile, Rachel suggested that governmental messaging was incoherent, which meant the most restrictive regulations were disproportionate:

Let’s say we sat here last Christmas and we said ‘right globally let’s make sure globally we stop everyone getting a cold and if anyone gets a cold society has let you down. You’ve let your mates down, your family down, your gran down’. It’s wrong this message. At the end of the day, it is a virus. There is also a broad spectrum to it. For some people it does nothing at all, some people are unwell, some people have a bad time, some people have to go to hospital while for others it is fatal. We have a better idea of high-risk categories but not everything. But to hold so many people hostage for an illness which is for them is inconsequential is something we need to think about. This is a pandemic. Historically in pandemics, people die. We need to protect the economy but it’s also important to look at things like how domestic violence has increased by 50% and there are children living in poverty without education who attend school for a safe space then have perhaps parents in their families losing jobs who start drinking and being violent. There is another generation of people we need to think about. (Rachel, NHS doctor)

Rachel’s point *returns* us to the Covid above all else strategy; the balance of harms presented by COVID-19; and the range of measures enacted by the government impact on NHS workers, whether it is COVID-19 admissions, domestic violence victims, those with postponed medical treatment for a wide range of chronic and deadly illnesses, or those displaying new and acute symptoms of mental ill health. NHS workers are reflective of the general population and thus there are diverse opinions and disagreement. Katie demonstrated frustration at the summer eat out to help out scheme, which is popularly believed to have caused the UK’s second wave. As Rachel also indicated, domestic violence rose in many nations across the world during the pandemic, particularly during the lockdowns. For instance, in the UK at the outset of the pandemic there was a 65% increase in calls to the national domestic abuse helpline, with many services reporting unprecedented demand (Healy et al., 2022). Such a positive motivation to harm was arguably the epidemic within the COVID-19 pandemic, with its effects reverberating long into the future. This includes further demand for services within the NHS, placing further pressure on a burnt out, exhausted, and under-resourced workforce.

## Conclusion

Our findings from a relatively small sample of NHS workers derived from online survey responses (N = 16), four online interviews, and one face-to-face interview (N = 5) offers a preliminary analysis of labouring in the NHS under conditions of crisis management and presents various issues that warrant further sociological research. The mode of working documented in the article amplified stress, fear, and anxiety that led to staff absence and other health problems. The absence of stability and protection is linked to workplace harm, including poor mental and emotional well-being. Public support and community spirit at the start of the pandemic gave way to antagonisms. People willing to break the lockdown rules to further their own expressive ends could be identified as a positive motivation to harm, which in some cases arguably manifested as special liberty (Hall, 2012). The article also documented the lack of PPE and the absence of coherent governmental messaging, with the former embodying a negative motivation to harm and amplifying the absence of protection for both NHS workers and patients. This aggravated fear and frustration in an already fractured neoliberalised social climate and workplace.

The macro-level of political economy and government policy impacts on the micro-level of working practice, and nowhere is this more evident than the impact of the pandemic upon the UK’s healthcare staff. Applying a social harm framework allowed us to consider the impact of the absence of protection and stability upon workers, the negative motivation to harm of austerity, as well as the special liberty associated with non-compliance. While a living with Covid strategy was announced in February 2022, the impact of the pandemic upon the NHS will be long-lasting with the harms upon both employees and patients continuing long into the future. At the time of writing (Winter 2022), staff shortages are pushing some NHS trusts to the brink of crisis. Ameliorating the post-pandemic NHS treatment backlog, which Bodapati et al. (2022) claim is *mission impossible* as it stands at 13 million people and will take 12 years to clear, will place profound pressure on the health service in the years ahead. As our article explicated, behind these statistics lies the toil, distress, and suffering of NHS workers who have laboured through a public health crisis on a scale unseen in modern times. This article presented some of the voices of those staff that experienced the harm of working in healthcare on the pandemic’s frontline.
